# A Molten Salt Extraction Method for Palladium Recovery via the CaPd_2_ Intermetallic Compound

**DOI:** 10.1002/advs.76629

**Published:** 2026-07-31

**Authors:** Jiapeng Zhang, Dengjie Yan, Muwen Chen, Baoqiang Xu, Bin Yang, Bingyi Song, Lingxin Kong

**Affiliations:** ^1^ Yunnan Key Laboratory of Non–ferrous Metals Vacuum Metallurgy Kunming University of Science and Technology Kunming China; ^2^ State Key Laboratory of Complex Non–Ferrous Metal Resources Clean Utilization Kunming University of Science and Technology Kunming China; ^3^ National Engineering Research Center of Vacuum Metallurgy Kunming University of Science and Technology Kunming China; ^4^ Faculty of Metallurgical and Energy Engineering Kunming University of Science and Technology Kunming China

**Keywords:** acid–free, CaPd_2_ intermetallic, first–principles calculation, molten salt extraction, zintl phase

## Abstract

To address palladium scarcity and the low efficiency of conventional recovery processes, this work presents a molten–salt extraction strategy for palladium recovery via the CaPd_2_ intermetallic compound. The process is based on the formation of CaPd_2_ and its subsequent oxidation by active iodine species generated in molten CaI_2_. First–principles calculations reveal a charge transfer of approximately 0.84 |e| from Ca to Pd in CaPd_2_, resulting in an ionic bonding framework with covalent contributions arising from Ca–Pd orbital hybridization. This unique bonding characteristic confers excellent thermal stability, with a mass loss of less than 0.13% up to 1273 K. The CaPd_2_ intermetallic compound was successfully synthesized and experimentally verified by X–ray diffraction and thermogravimetric analysis. In molten CaI_2_, iodine species selectively oxidize the anionic Pd species in CaPd_2_, enabling the direct recovery of metallic Pd. Under optimized conditions (1073 K, 1 h), palladium with a purity of 99.5 ± 0.3 wt.% was obtained. Unlike conventional pyrometallurgical and hydrometallurgical processes, the proposed approach eliminates the acid–leaching step and achieves rapid Pd recovery within 1 h. The results establish an iodine–mediated molten–salt recovery mechanism and provide a potential route for future palladium recovery from secondary resources.

## Graphical abstract

A designed Ca → CaPd_2_ → Pd pathway enables acid–free palladium recovery in molten salt. Calcium alloying forms anionic CaPd_2_, which is selectively oxidized by iodine from CaI_2_ to yield high–purity metallic Pd.

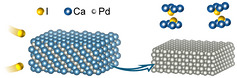

## 1 Introduction

**FIGURE 1:**
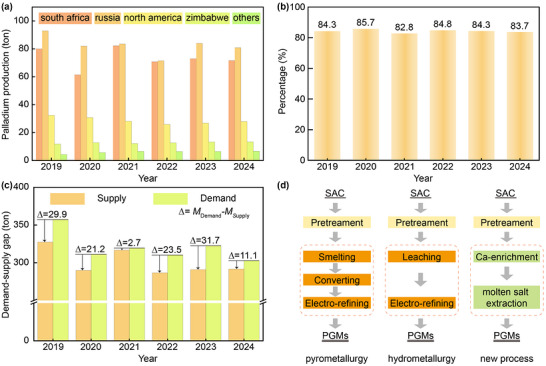
Global Pd metal production and consumption: (a) annual production in various countries, (b) percentage demand from different fields, (c) changes in supply and demand, and (d) schematic illustration of a potential application scenario for Pd recovery from spent catalysts, adapted from ref. [25].

As a geologically scarce metal [1], palladium (Pd) has critical applications in the electronics, chemical manufacturing, and automotive fields [2]. The unique physicochemical properties of palladium enable its widespread technological and industrial applications [3, 4, 5, 6, 7, 8]. Palladium resources are geographically concentrated (Figure 1a), and the demand from the catalyst industry is strong (Figure 1b). However, recycling from end–of–life catalysts is minimal (Figure 1c) [9]. Among the PGMs, Pd dominates catalytic applications across industries. Thus, recycling Pd from spent catalysts is essential for the sustainable utilization of Pd resources [10].

Pd catalysts can be classified into four types based on the support material: Pd/C, Pd/Al_2_O_3_, Pd/SiO_2_, and Pd/cordierite [11, 12, 13, 14, 15, 16, 17, 18, 19]. Automotive three–way catalysts, the most abundant palladium source [20], contain Pt and Pd as primary PGMs [21]. The recovery of Pd from spent catalysts comprises three key stages: pretreatment, crude extraction, and refining [22, 23]. Established methods include pyrometallurgical and hydrometallurgical approaches, while emerging techniques, such as polymer–assisted supercritical CO_2_ extraction, enable the recovery of metals under mild conditions [24]. However, pyrometallurgical and hydrometallurgical methods suffer from low dissolution efficiencies, multiple separation steps, and hazardous liquid waste generation in large amounts [25]. Figure 1d illustrates a conceptual recovery route adapted from the literature and is presented only to provide the broader application context. The Ca–enrichment step has not been experimentally investigated in the present work.

Flandinet pioneered molten salt dissolution for metal recovery from waste plastics to address hazardous waste generation [26]. Building on this foundation, we propose a calcium–alloying strategy for palladium recovery, which converts Pd into Ca–Pd compounds with enhanced dissolution kinetics and anionic stability [27]. A stable Ca–Pd intermetallic compound was prepared by rationally exploiting the high reactivity of calcium. Pd anions undergo rapid oxidation to metallic palladium by molten salt–derived iodine, achieving complete reaction within 1 h. Unlike prior approaches that rely on acid leaching, this method employs a molten CaI_2_ medium where in situ generated iodine selectively oxidizes anionic Pd in CaPd_2_ to metallic Pd^0^. This acid–free process achieves 99.5 ± 0.3 wt.% Pd purity within 1 h at 1073 K, eliminating both acid consumption and hazardous liquid waste (Table S2) [28]. Specifically, it is hypothesized that CaPd_2_ can serve as an intermediate phase in which Pd exists in an anionic state and can be selectively oxidized by iodine species generated in molten CaI_2_, thereby enabling efficient palladium recovery without conventional acid leaching.

## 2 Results and Discussion

### 2.1 Electronic Structure of CaPd_2_

CaPd_2_ adopts a cubic Laves phase structure that is isotypic to that of MgCu_2_ ($Fd\bar{3}m$, No.227) [29]. In this AB_2_–type compound, each calcium atom is coordinated by 12 equivalent palladium atoms (CN = 12), while each palladium atom is coordinated by 12 calcium atoms and 4 palladium atoms (CN = 16), forming a 3D network of corner– and edge–sharing polyhedra. The Pd–Pd bond length is 2.72 Å, and the Ca–Pd bond length is 3.18 Å, significantly shorter than the sum of the van der Waals radii of Ca and Pd (∼5.13 Å) [30], indicating metallic bonding. The cell parameters and atomic coordinates are listed in Table S1.

**FIGURE 2:**
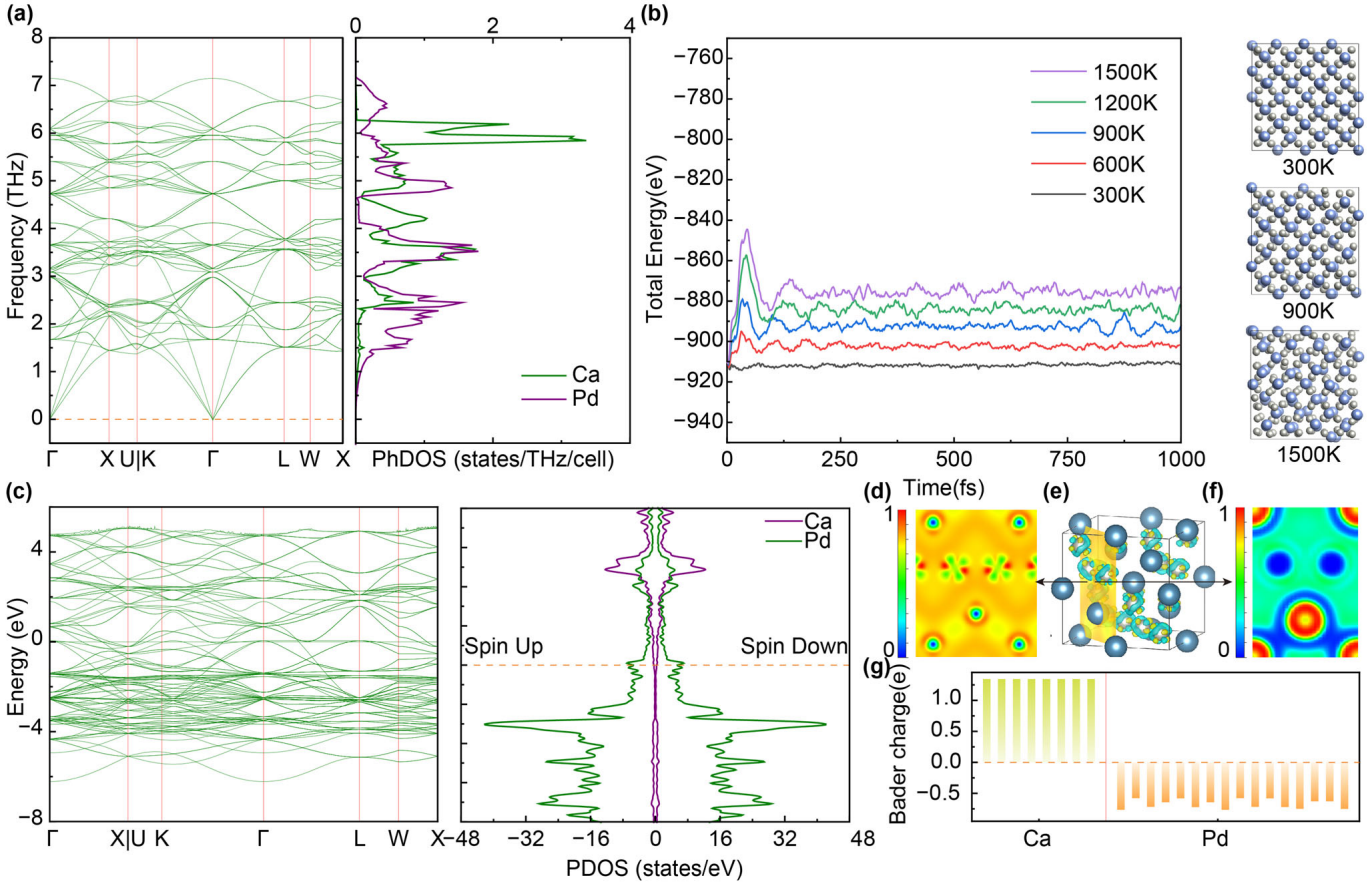
Bonding properties of CaPd_2_: (a) phonon dispersion (left) and Ca/Pd–projected phonon density of states (PhDOS) (right), (b) temperature–dependent total energy profiles and snapshots of molecular dynamics simulations at 300, 900, and 1500 K (side view) after 1 ps of simulated annealing, (c) electronic band structure and density of states of Ca/Pd atom, (d) and (e) charge–density difference with an isosurface value of 0.01 e Å^−3^ (yellow: charge enrichment; blue: charge loss), (f) ELF isosurface map, and (g) Bader charge analysis.

The phonon dispersion of the CaPd_2_ crystal (Figure 2a) reveals the absence of an imaginary frequency, confirming the dynamic stability of its lattice. The phonon density of states (PhDOS) indicates that both Ca and Pd atoms contribute across the entire frequency range, reflecting the strongly coupled lattice dynamics of this intermetallic compound. In the low–frequency acoustic vibration region (below ∼2.5 THz), the vibrational modes show a relatively large contribution from Pd atoms, whereas Ca and Pd atoms exhibit comparable contributions in the optical frequency range, indicating significant mode mixing between the two atomic species. Such mixed vibrational characteristics are typical of Laves phase intermetallic compounds, in which the atomic mass effects are partially compensated by the comparable interatomic force constants associated with metallic bonding. Figure 2b shows the total energy evolution as a function of the simulation time obtained from molecular dynamics simulations at 300, 900, and 1500 K, together with representative structural snapshots after 2 ps of simulated annealing, demonstrating the thermal stability of CaPd_2_. The electronic band structure of CaPd_2_ (Figure 2c) shows multiple bands crossing the Fermi level (*E*_F_), which is indicative of its metallic nature and pronounced band hybridization. Several weakly dispersed bands near the *E*_F_ predominantly originate from the Pd *d* states, which dominate the electronic structure below the *E*_F_. The projected density of states (PDOS) exhibit nearly identical spin–up and spin–down components, confirming the nonmagnetic ground state of CaPd_2_ and its metallic character with a finite density of states at the *E*_F_. The differential charge density maps (Figure 2d,e) reveal a clear electron transfer from Ca atoms with lower electronegativity to Pd atoms with higher electronegativity. This charge redistribution is further supported by the electron localization function (ELF) analysis (Figure 2f), which indicates enhanced electron localization around the Pd atoms compared to that around the Ca atoms. These results indicate the mixed metallic–ionic bonding characteristic of CaPd_2_. Bader charge analysis (Figure 2g) yielded net charges of approximately +0.84 |e| for Ca and −0.42 |e| for Pd atoms, satisfying charge neutrality in CaPd_2_ [31]. Such charge transfer highlights the strong electronic interactions between the Ca and Pd atoms and underscores the important role of the local electronic environment in governing the bonding characteristics of Pd in this intermetallic compound.

**FIGURE 3:**
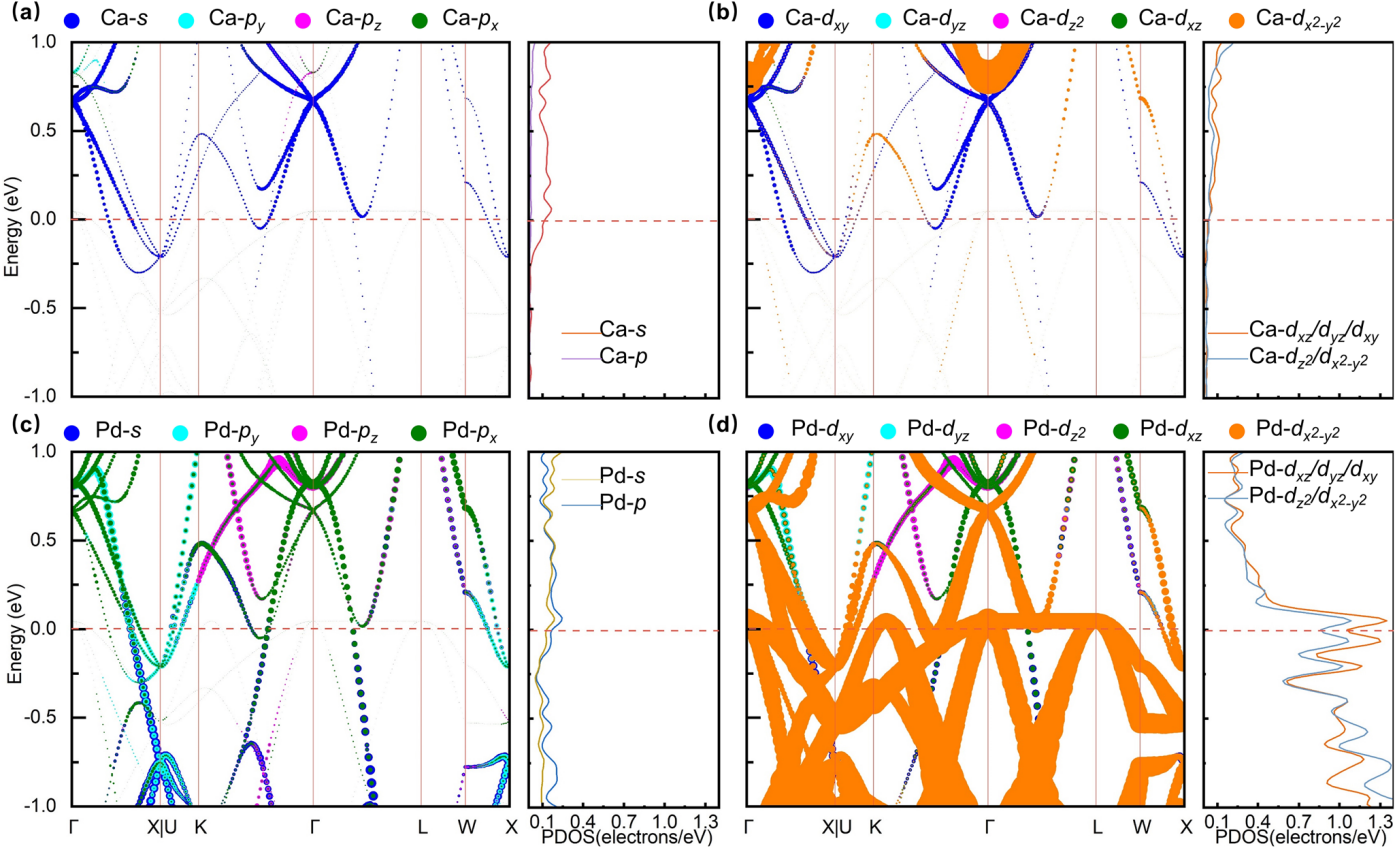
Atom–orbital–resolved band structure and density of states of intermetallic CaPd_2_: (a) Ca–*s* and Ca–*p_x_/p_y_/p_z_*, (b) Ca–*d_xz_/d_yz_/d_xy_* and Ca–*d_z_* ^2^ */d_x_* ^2^ *_–y_* ^2^, (c) Pd‐*s* and Pd–*p_x_/p_y_/p_z_*, and (d) Pd–*d_xz_/d_yz_/d_xy_* and Pd–*d_z_* ^2^ */d_x_* ^2^ *_–y_* ^2^.

Figure 3 shows the atom– and orbital–resolved band structures and density of states of CaPd_2_. Palladium exhibits a partially filled *d–*manifold near the *E*_F_, indicating its tendency to accept electrons from neighboring atoms. The PDOS reveal significant hybridization between the *s* orbital of Ca and the *d* orbital of Pd in the energy range of approximately −1 to 0 eV relative to the *E*_F_, suggesting covalent interaction between Ca and Pd.

### 2.2 Experimental Synthesis and Molten Salt Extraction of Pd from CaPd_2_

**FIGURE 4:**
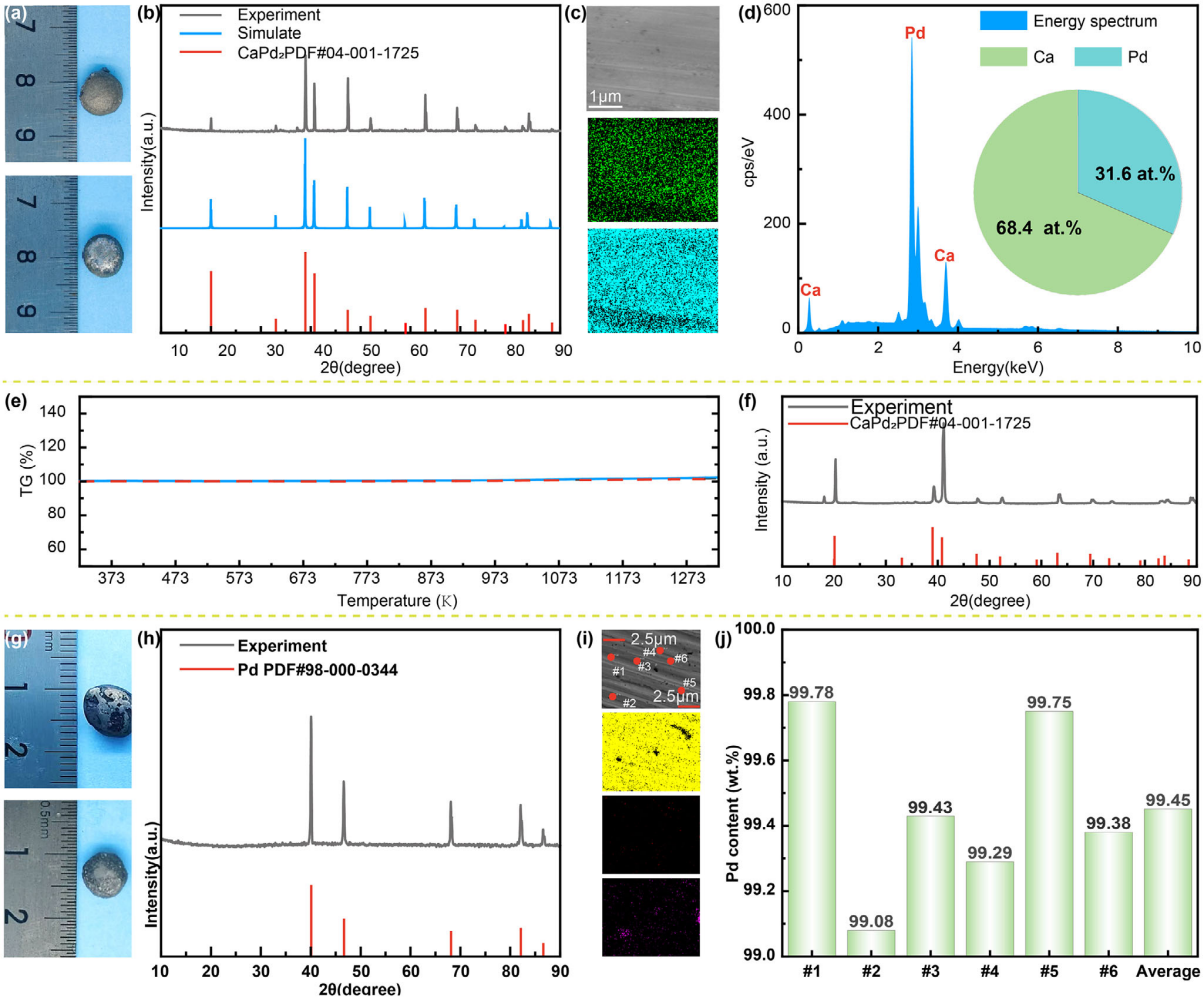
Physical phase analysis of CaPd_2_ samples: (a) macroscopic morphology, (b) experimental XRD pattern, along with simulated and standard powder diffraction file (PDF) patterns, (c) SEM image before dissolution, (d) semi–quantitative EDS analysis before dissolution, (e) TGA curves (blue line: 9.08 mg, red line: 24.098 mg), (f) post–TGA XRD patterns, (g) sample before and after dissolution, (h) XRD pattern after dissolution, (i) SEM image after dissolution, and (j) palladium content at six random measurement points in the SEM image.

Figure 4a shows a photograph of the synthesized CaPd_2_ specimen. The experimental X–ray diffraction (XRD) pattern was consistent with the pattern obtained by density functional theory (DFT) simulations and the standard pattern in the inorganic crystal structure database (ICSD) (Figure 4b). The characteristic peaks observed at 20.1° (111), 39.0° (311), and 40.8° (222) confirm a cubic phase. The refined lattice parameters, *a* = 7.652 Å (*α* = *β* = *γ* = 90°) exhibit less than 0.4% deviation from the $Fd\bar{3}m$ theoretical values, validating the structural model. Scanning electron microscopy (SEM) data confirmed the uniform granularity and absence of discernible defects in the CaPd_2_ specimen (Figure 4c). Semi–quantitative energy dispersive X–ray spectroscopy (EDS) mapping revealed a Ca:Pd atomic ratio of 31.6:68.4, consistent with the nominal CaPd_2_ stoichiometry (Figure 4d). The combination of XRD and EDS analyses validated the near–stoichiometric composition of the arc–melted CaPd_2_.

Figure 4e shows the thermogravimetric analysis (TGA) profile of CaPd_2_ (Δ*m* < 0.13% at 1273 K), confirming its thermal stability against decomposition, oxidation, and volatilization. Post–TGA XRD analyses (Figure 4f) confirm that the crystalline structure was retained with consistent peak positions and intensities, and no impurity phases or lattice strains were observed. The surface morphology indicated the absence of melting or phase segregation, thereby validating the thermal stability of CaPd_2_.

CaPd_2_ exhibited a compact and smooth surface before dissolution and underwent morphological alterations with size reduction after dissolution (Figure 4g). XRD analysis after dissolution (Figure 4h) confirmed the disappearance of the diffraction peaks of CaPd_2_ and the appearance of the peaks of elemental palladium. The corresponding SEM results (Figure 4i) revealed that only Pd was present in the dissolved residue, whereas Ca remained in a discrete phase before the post–treatment stage. However, at this stage, Ca and Pd did not undergo alloying. EDS spot analysis (Figure 4j) provided a semi–quantitative estimate of the palladium purity, yielding 99.5 ± 0.3 wt.% with residual calcium (0.16 wt.%) and iodine (0.39 wt.%).

Furthermore, time–resolved experiments were conducted over a range of reaction times (0.25 to 2 h) under identical conditions (1073 K). XRD analysis (Figure S1) confirmed complete conversion in all cases, with only metallic Pd peaks detected at every time point, demonstrating the high reproducibility of the process.

### 2.3 Mechanism of Molten Salt Extraction of Pd from CaPd_2_

**FIGURE 5:**
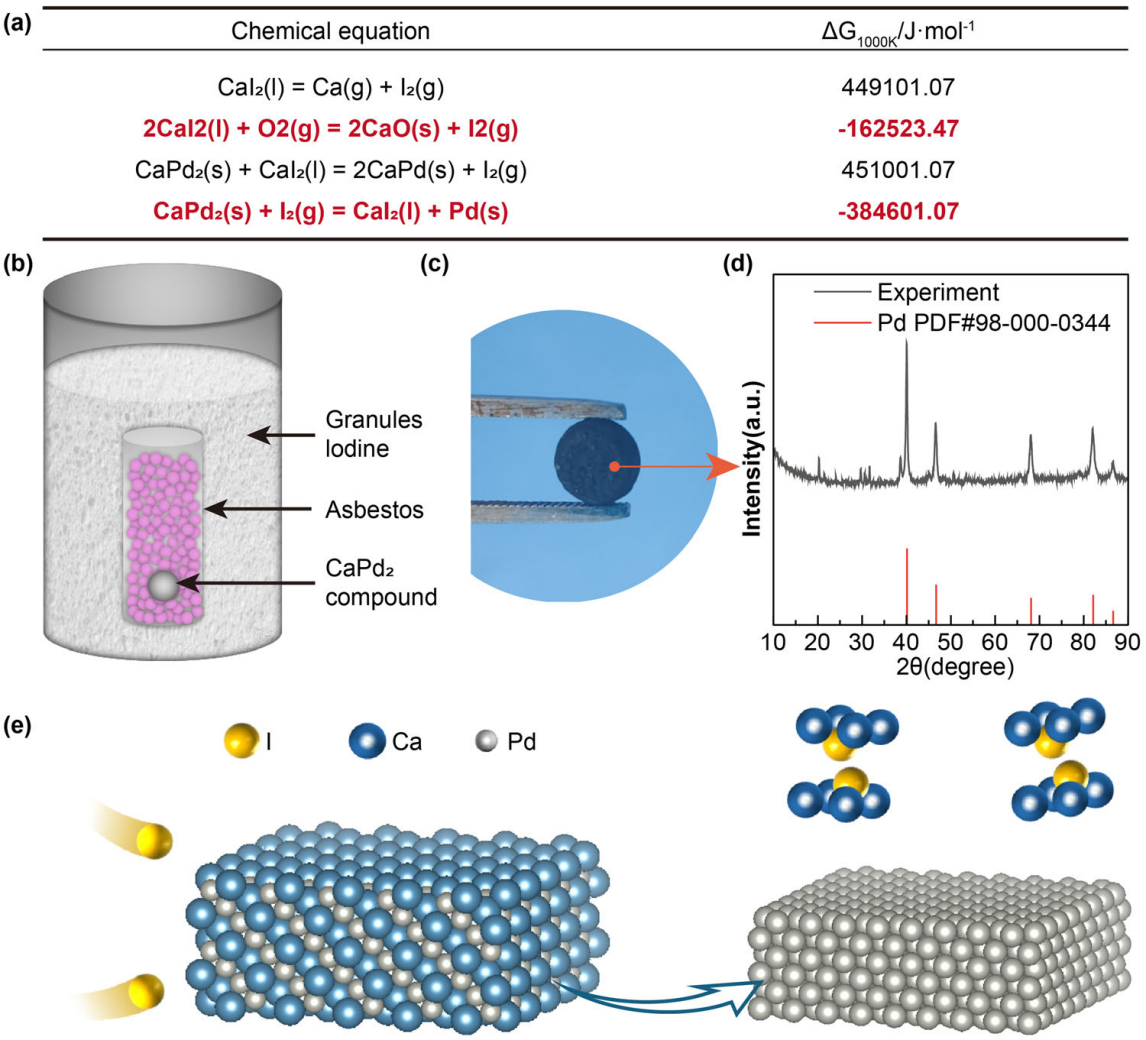
Reaction of I_2_ with CaPd_2_: (a) mechanism of iodine displacement from CaPd_2_, (b) schematic of the reactor assembly, (c) optical photograph of the reaction products, (d) XRD patterns, and (e) reaction mechanism.

Figure 5a presents the thermodynamic calculations for the CaPd_2_ reactions in molten CaI_2_ at 1000 K using the data available in literature [32, 33]. A previous study confirmed that the fourth reaction contributes to the stability of CaPd_2_ at 1000 K [34]. Gibbs free energy (Δ*G*) analysis indicated that CaI_2_ reacts with trace oxygen to generate iodine vapor, consistent with our experimental observations. The released iodine vapor further oxidizes the Pd ions to elemental Pd [35]. Wannek demonstrated the iodine–catalyzed synthesis of Mg–Pd and Al–Pd intermetallic compounds [36, 37] and confirmed the higher stability of these phases relative to those of their iodides (MgI_2_ and AlI_3_, respectively). The thermodynamic data confirmed the superior stability of CaI_2_ relative to that of CaPd_2_, driving the spontaneous iodine–induced Pd displacement, resulting in metallic Pd extraction from CaPd_2_.

In the double–crucible setup (Figure 5b), 0.5 g of CaPd_2_ was reacted with 10 g of iodine (99.99%) under a titanium–sponge oxygen barrier at 473 K for 10 h. Post–reaction analyses revealed white deposits on the surface and an inner black powder (Figure 5c); the phase composition of the latter is shown in Figure 5d. Physical phase analysis confirmed that the surface substitution between iodine and CaPd_2_ at 473 K yielded metallic Pd (as evidenced by the Pd(111)/(200) XRD peaks). Solid–solid mass–transfer limitations confine the reaction to the surface layer, resulting in a significant loss of volatilized iodine. Electronic structure analysis elucidates the mechanistic origin of this efficient extraction. Bader charge analysis confirms that Pd in CaPd_2_ carries a partial negative charge, indicating an electron–rich anionic state. This anionic Pd is thermodynamically more susceptible to oxidation by iodine than neutral Pd^0^, enabling rapid and selective oxidative decalcification in molten CaI_2_ without strong acids. Employing the CaI_2_ molten salt system (melting point: 1052 K) shifts the reaction to the solid–liquid mode, enhancing mass transfer via dissolved iodine. The argon flow serves to carry excess iodine vapor into the off–gas treatment system for safe absorption. The instant dissolution of the calcium monomers disrupts the equilibrium, resulting in the Pd purity to 99.5 ± 0.3 wt.% as estimated by semi–quantitative EDS analysis, significantly upgrading the solid–solid reaction outcome (Figure 5e). Notably, the calcium initially used for Pd capture is ultimately converted to CaI_2_ during the oxidative decalcification step. This CaI_2_ serves as the molten salt medium and can be recycled, closing the material loop.

## 3 Conclusion

We presented a new strategy for sustainable Pd recovery. A CaPd_2_ intermetallic compound was formed through Ca alloying, and a molten salt dissolution method was employed to selectively remove calcium to extract Pd. Electronic and thermodynamic analyses confirmed the anionic nature of Pd in CaPd_2_ and provided a theoretical basis for the oxidation of Pd anions to metallic Pd using iodine. Molten salt decomposition at high temperatures releases iodine, which oxidizes Pd*^δ−^* to Pd^0^, enabling its efficient recovery. When heated at 1073 K in molten CaI_2_, CaPd_2_ undergoes selective decalcification, yielding metallic Pd with a mean purity of 99.5 ± 0.3 wt.%. This method offers significant advantages, such as efficient extraction (0.5–1 h) and no acidic waste. As a mechanistic study, the present work focuses on validating the core chemistry of the proposed pathway, providing guidance for future palladium recovery from real spent catalysts.

## 4 Methods

### 4.1 Theoretical Calculations

Spin–polarized DFT calculations were performed using Vienna Ab initio Simulation Package (VASP) [38, 39] to optimize the CaPd_2_ crystal structures and evaluate their electronic properties [40, 41, 42, 43]. The projector–augmented wave (PAW) method was used to describe electron–ion interactions, and the Perdew (Burke) Ernzerhof generalized gradient approximation (GGA) was employed for the exchange–correlation functional. Valence electrons were treated in the configurations of Ca–3s^2^3p^6^4s^2^ and Pd–4d^9^5s^1^, as provided in the VASP PAW potentials. A plane–wave energy cutoff of 400 eV and a Γ–centered Monkhorst–Pack k–point mesh of 7 × 7 × 7 were used.

The phonon dispersion of the CaPd_2_ compound was calculated using the finite displacement method implemented in the Phonopy code [44]. To obtain reliable phonon dispersion results, more accurate DFT calculations were performed for further geometric optimization accuracy. The more stringent energy convergence criteria were set to 500 eV for the cutoff energy, 10^−7^ eV for the total energy, and 10^−2^ eV/Å for force convergence during the phonon calculations. Ab initio molecular dynamics (AIMD) simulations were conducted in the canonical ensemble (NVT) at the GGA–Perdew–Burke–Ernzerhof (PBE) level to assess the structural stability at several temperatures. Bader charge and charge density difference (CDD) analyses were performed to investigate the charge–transfer mechanism, which was further interpreted using the PDOS [45, 46]. The crystal structures and electronic properties were visualized and analyzed using VESTA and Multiwfn software [47, 48, 49].

### 4.2 Preparation of CaPd_2_ Intermetallic Compounds

Ca–Pd intermetallics were synthesized in an argon–protected arc furnace (≤1 Pa vacuum, Shenyang Kejing, China). After the calcium particles (purity ≥ 95%, Yunnan Keyi, China) were loaded and uniformly coated with the palladium powder (purity ≥ 99.999%, Beijing Zhongnuo, China), the chamber was evacuated to <1 Pa, backfilled with 99.999% argon, and triple–purged to minimize the O_2_/H_2_O content. Arc melting was performed at 110 A for 4 min in pulsed mode (0.5 s cycle, 50% duty), with the spray gun centered 2 cm above the sample crucible. Real–time observations were made to ensure uniform melting through dynamic adjustments. Under high–temperature melting, Ca and Pd easily form ordered surface compounds [50], and the intermediate phases of Ca_9_Pd (melting point: 938 K), Ca_3_Pd (melting point: 843 K), and Ca_5_Pd_2_ (melting point: 868 K) decompose before reaching their melting points [51]. Repeated melting converts CaPd into CaPd_2_, ultimately providing stable CaPd_2_ (melting point: 1573 K) [34].

### 4.3 Dissolution Experiment on CaPd_2_ Intermetallic Compounds

The pretreated CaI_2_ (purity ≥ 99%, Wuhan Huaxiang, China) molten salt was dehydrated at 473 K in a vacuum oven for 12 h and transferred to an alumina crucible in the reactor. The reactor was then sealed and heated to 1073 K at a rate of 10 K/min under a continuous argon flow, and maintained at this temperature for 0.5–1 h after stabilization. The argon flow served to carry excess iodine vapor into the off–gas treatment system. Thereafter, the prepared CaPd_2_ was added to the molten salt. After dissolution for 1 h, the alloy residue was extracted using a BN sampler and washed with ethylenediaminetetraacetic acid to remove the molten salt residue and I_2_ from the metal surface [52]. The samples were dried at 473 K in a vacuum oven for 2 h, vacuum sealed, and transferred to a glove box.

### 4.4 Sample Material Characterization

The synthesized CaPd_2_ was analyzed using XRD (X'Pert3 Powder diffractometer, Malvern Panalytical, UK; Cu *Kα* radiation, *2θ* range of 10°–90°, step size of 0.02626°).

SEM–EDS was used to characterize the elemental distribution, surface morphology, and atomic composition of the dissolved metals via semi–quantitative analysis.

The thermal stability of CaPd_2_ was characterized through TGA in an Ar atmosphere (293–1373 K at 20 K/min), and the mass was monitored continuously.

## Author Contributions

Lingxin Kong, Bin Yang and Baoqiang Xu conceived the idea, supervised the progress of the project, and revised the manuscript. Jiapeng Zhang, Dengjie Yan, and Muwen Chen carried out experiments and first–principles structural calculations, and wrote the paper. All authors contributed to this work and agreed to the entire contents of this manuscript.

## Conflicts of Interest

The authors declare no conflict of interest.

## Supporting information




**Supporting File 1**: advs76629‐sup‐0001‐SuppMat.docx.

**Supporting File 2**: advs76629‐sup‐0002‐Data.zip.

## Data Availability

The data that support the findings of this study are available in the supplementary material of this article.
