# Supplementary material for: A Molten Salt Extraction Method for Palladium Recovery via the CaPd2 Intermetallic Compound
Source: Adv Sci (Weinh). 2026 Jul 31:e76629. Online ahead of print. doi: 10.1002/advs.76629 (PMC13427229; doi:10.1002/advs.76629)
Supplement: Supplementary file 1 — Supporting File 1: advs76629‐sup‐0001‐SuppMat.docx. [file ADVS-9999-e76629-s001.docx]

# **Table S1 Crystal structures and atomic coordinates of CaPd_2_ compounds**

| **Space group** | **Lattice Parameters(Å)** | **Atom** | **Atomic positions (fractional)** |
| --- | --- | --- | --- |
| Fd-3m | a = 7.67928 | Ca1 | 0.75000 0.25000 0.25000 |
|  | b = 7.67928 | Ca2 | 0.00000 0.00000 0.50000 |
|  | c = 7.67928 | Ca3 | 0.75000 0.75000 0.75000 |
|  | α = 90° | Ca4 | 0.00000 0.50000 0.00000 |
|  | β = 90° | Ca5 | 0.25000 0.25000 0.75000 |
|  | γ = 90° | Ca6 | 0.50000 0.00000 0.00000 |
|  |  | Ca7 | 0.25000 0.75000 0.25000 |
|  |  | Ca8 | 0.50000 0.50000 0.50000 |
|  |  | Pd1 | 0.87500 0.12500 0.87500 |
|  |  | Pd2 | 0.87500 0.87500 0.12500 |
|  |  | Pd3 | 0.12500 0.87500 0.87500 |
|  |  | Pd4 | 0.12500 0.62500 0.62500 |
|  |  | Pd5 | 0.87500 0.62500 0.37500 |
|  |  | Pd6 | 0.87500 0.37500 0.62500 |
|  |  | Pd7 | 0.12500 0.37500 0.37500 |
|  |  | Pd8 | 0.12500 0.12500 0.12500 |
|  |  | Pd9 | 0.37500 0.12500 0.37500 |
|  |  | Pd10 | 0.37500 0.87500 0.62500 |
|  |  | Pd11 | 0.62500 0.87500 0.37500 |
|  |  | Pd12 | 0.62500 0.62500 0.12500 |
|  |  | Pd13 | 0.37500 0.62500 0.87500 |
|  |  | Pd14 | 0.37500 0.37500 0.12500 |
|  |  | Pd15 | 0.62500 0.37500 0.87500 |
|  |  | Pd16 | 0.62500 0.12500 0.62500 |

**Table S2. Comparison of key process metrics for different Pd recovery methods.**

| **Dimension** | **This work** | **Hydrometallurgy** | **Pyrometallurgy** |
| --- | --- | --- | --- |
| **Temperature (°C)** | 800 | 60–120 | 1300–1600 |
| **Main reagents** | Ca metal;  CaI_2_ (recyclable) | Strong acids;  oxidants | Metal collectors; silica/fluxes |
| **Liquid waste** | Minimal | Large | Negligible |
| **Gas emission** | Negligible | Cl_2_, NO*_x_* | CO_2_, SO*_x_* |
| **Solid residue** | Recyclable CaI_2_ salt | Neutralization sludge | Smelting slag |


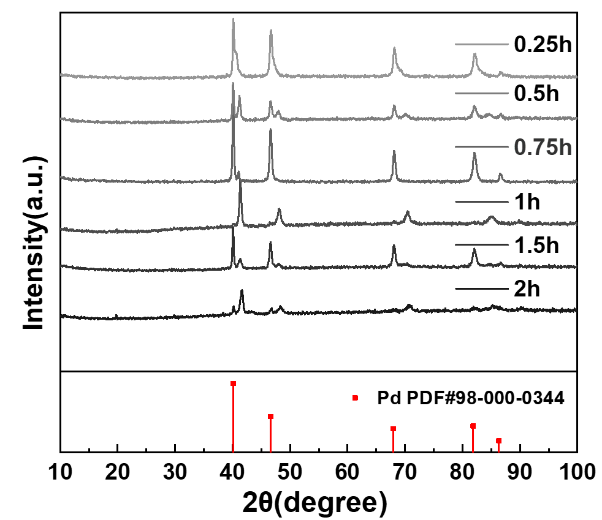


**Figure S1. Time-resolved XRD patterns of the reaction products at different time intervals (0.25–2 h) under 1073 K.**
